# Meta-analysis of host transcriptional responses to SARS-CoV-2 infection reveals their manifestation in human tumors

**DOI:** 10.1038/s41598-021-82221-4

**Published:** 2021-01-28

**Authors:** Fengju Chen, Yiqun Zhang, Richard Sucgang, Sasirekha Ramani, David Corry, Farrah Kheradmand, Chad J. Creighton

**Affiliations:** 1 Dan L. Duncan Comprehensive Cancer Center Division of Biostatistics, Baylor College of Medicine, Houston, TX 77030 USA; 2 Department of Molecular Virology and Microbiology, Baylor College of Medicine, Houston, TX 77030 USA; 3 Center for Translational Research in Inflammatory Diseases, Michael E. DeBakey VA, Houston, TX 77030 USA; 4 Departments of Pathology and Immunology, Baylor College of Medicine, Houston, TX 77030 USA; 5 Biology of Inflammation Center, Baylor College of Medicine, Houston, TX 77030 USA; 6 Department of Medicine, Baylor College of Medicine, Houston, TX 77030 USA; 7 Department of Bioinformatics and Computational Biology, The University of Texas MD Anderson Cancer Center, Houston, TX 77030 USA; 8 Human Genome Sequencing Center, Baylor College of Medicine, Houston, TX 77030 USA

**Keywords:** Cancer, Lung cancer, Tumour immunology, Infection, Inflammation, Data integration, Gene regulatory networks

## Abstract

A deeper understanding of the molecular biology of SARS-CoV-2 infection, including the host response to the virus, is urgently needed. Commonalities exist between the host immune response to viral infections and cancer. Here, we defined transcriptional signatures of SARS-CoV-2 infection involving hundreds of genes common across lung adenocarcinoma cell lines (A549, Calu-3) and normal human bronchial epithelial cells (NHBE), with additional signatures being specific to one or both adenocarcinoma lines. Cross-examining eight transcriptomic databases, we found that host transcriptional responses of lung adenocarcinoma cells to SARS-CoV-2 infection shared broad similarities with host responses to multiple viruses across different model systems and patient samples. Furthermore, these SARS-CoV-2 transcriptional signatures were manifested within specific subsets of human cancer, involving ~ 20% of cases across a wide range of histopathological types. These cancer subsets show immune cell infiltration and inflammation and involve pathways linked to the SARS-CoV-2 response, such as immune checkpoint, IL-6, type II interferon signaling, and NF-κB. The cell line data represented immune responses activated specifically within the cancer cells of the tumor. Common genes and pathways implicated as part of the viral host response point to therapeutic strategies that may apply to both SARS-CoV-2 and cancer.

## Introduction

Worldwide, the Coronavirus disease 19 (COVID-19) pandemic has resulted in over 18 million confirmed cases and over 700,000 deaths^1^, with government-enforced mitigation measures, including lockdowns, likely to have widespread socio-economic effects, both short term and long term^2^. While a sizeable proportion of COVID-19 patients are asymptomatic or have only mild symptoms, other cases range from moderate to severe or critical^3^, with the most vulnerable patients being those of advanced age or with underlying health conditions such as diabetes mellitus, chronic lung disease, or cardiovascular disease^4^. COVID-19 is caused by the severe acute respiratory syndrome coronavirus 2 (SARS-CoV-2). The hallmark of lower respiratory tract beta coronavirus family, including SARS-CoV-2, is viral pneumonia accompanied by systemic inflammation, respiratory failure, and acute respiratory distress syndrome (ARDS)^5,6^. The host response to a virus is generally not uniform, and infections can, therefore, inflict different degrees of morbidity and mortality^7^. We need a deeper understanding of how SARS-CoV-2 infects cells and how the host responds to the virus.

Recently, gene transcription profiling studies of model systems or human patient samples involving SARS-CoV-2 infection have been reported^7,8^. With the associated molecular data available in the public domain, the host transcriptional response of SARS-CoV-2, as observed in different cellular contexts, may be defined and compared with the host responses of other respiratory viruses. For example, in a recent study^7^, the host response in lung cell lines was defined by elevated chemokines and high expression of IL-6 and by low levels of type I and III interferons. Host responses, as observed using experimental models (e.g., in vitro cell lines), may be compared with molecular data from COVID-19 patients, to identify genes commonly altered in both settings. The available data also allow us to identify host responses common across different viruses and host responses specific to coronaviruses, including SARS-CoV-2.

Transcriptional data on host responses of cancer cells to SARS-CoV-2 infection in vitro also provide an intriguing opportunity to compare these data with human tumor data. Approximately 10% of all cancers are linked to viruses, whereby some (e.g., human papillomavirus) drive a malignant phenotype by continuous viral oncogene expression or modification of host genes^9^. Beyond the above, there could conceivably be parallels between the transcriptional program initiated in response to viral infection and how tumors interact with the immune system. Both microbes and tumors activate innate resistance, tissue repair, and adaptive immunity^10^. To the immune system, cancer cells may present as infected cells, as they express aberrant proteins as surface antigens^11^, which anomalies are also a common feature of viral and other infections. Therefore, immune system evasion represents a hallmark of cancer, as manifested in the alteration of immune checkpoint pathways^12^. Cancer is a heterogeneous disease, with different sets of somatic genomic alterations that confer uncontrolled cellular proliferative capacity^13,14^. There are multiple mechanisms for a cancer-mediated escape from immune responses, including usurping pathways shared by the viral host response^10,15^. Host responses to infection can differ between different cancer cell lines, which may reflect distinct ways in which these cancers have evolved throughout the disease.

In this present study, we defined transcriptional gene signatures of the host response to SARS-CoV-2 infection, using three different lung cell lines, one normal epithelial cell line and two lung adenocarcinoma cell lines. We identified signatures common across all three cell lines and signatures specific to one or both adenocarcinoma lines. We found significant overlaps between the SARS-CoV-2 in vitro signatures with genes associated with human COVID-19 infections in tracheal samples. Using the SARS-CoV-2 signatures, we probed multiple gene expression profiling datasets from independent studies involving infection by other viruses in different model systems and human patient data. We found broad similarities between the host responses to SARS-CoV-2 with that of other viruses. Finally, we discovered that the SARS-CoV-2 transcriptional signatures also manifested within specific subsets of human cancer. These subsets were identified previously using class discovery^14,16^ and involved the immune response and immune checkpoint pathway, as well as IL-6, type II interferon signaling, and NF-Kappa B (NF-κB) pathways.

## Results

### Transcriptional signatures of the host response to SARS-CoV-2 infection in human bronchial epithelial cells and lung cancer cell lines

**Figure 1:**
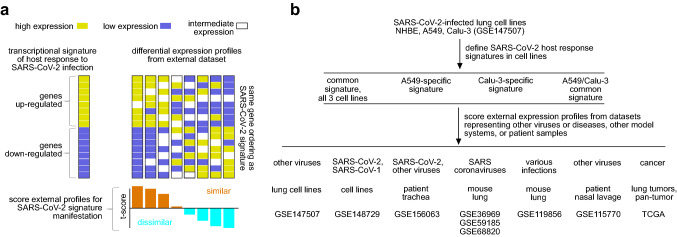
Overview of the basic approach of the study. (**a**) Diagram of the overall analytical approach to score a set of differential expression profiles according to a given gene transcription signature. For a given expression dataset, we score each mRNA profile according to an independently-derived transcriptional signature representing the host response to SARS-CoV-2 infection. The scoring basis is on whether the relative differential patterns in the external sample profile, higher versus lower, are broadly similar to the patterns of up- versus down-regulation, respectively, in the SARS-CoV-2 signature. The “t score” signature scoring metric from previous studies is used^34–37^. (**b**) Diagram of the study. We first defined transcriptional gene signatures of SARS-CoV-2 infection, using three different lung cell lines (NHBE, A549, Calu-3)^7^. We then applied these signatures to examine differential expression profiles from the indicated external transcriptome datasets representing other viruses or diseases, other model systems, or patient samples.

**Figure 2:**
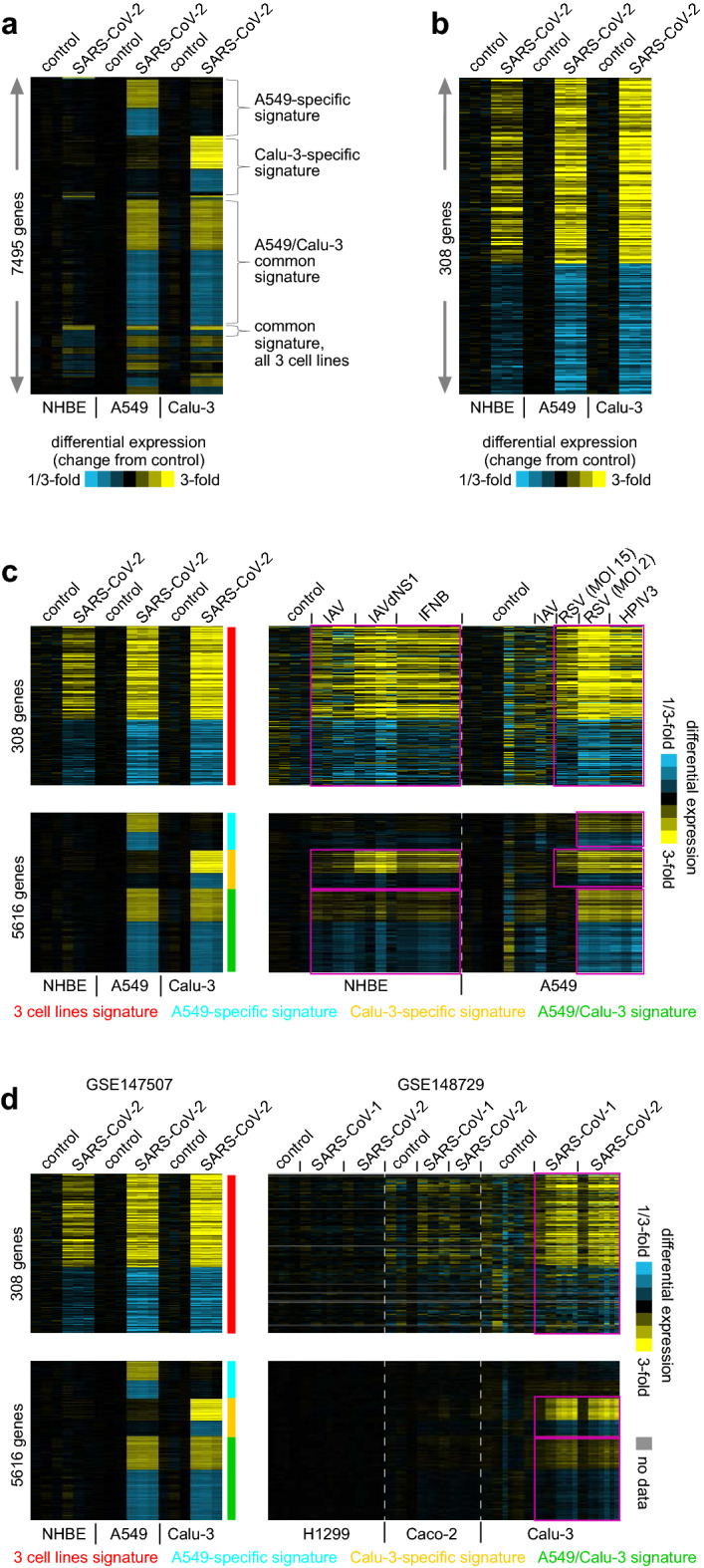
Transcriptional signatures of the host response to SARS-CoV-2 infection across three different human lung cell lines. (**a**) Three different human lung cell lines (NHBE, A549, Calu-3) were infected with SARS-CoV-2 at multiplicity-of-Infection (MOI) of 2 and profiled for gene expression by RNA-seq (GSE147507^7^). Genes altered with p < 0.01 for any cell line are represented as a heat map. Each cell line profile is centered on the average of its corresponding mock control group. (**b**) Heat map representing a common set of 308 genes up-regulated or down-regulated across all three cell lines and in the same direction of change (one-sided p < 0.05 NHBE, and two-sided p < 0.01 A549 and Calu-3). (**c**) In the same study noted above, NHBE or A549 cells were infected with other viruses (IAV, RSV, HPIV3) or treated with interferon beta (IFNB). Differential SARS-CoV-2-associated expression patterns common to all three cell lines (NHBE, A549, Calu-3) or found for just one or two cell lines (taken from **a**) are shown for both the SARS-CoV-2 infection profiles and the additional profiles representing the other infections and treatments. Each treatment profile is centered on the average of its corresponding control group. Patterns of manifestation of SARS-CoV-2 signatures within the other virus or treatment groups are highlighted. (**d**) In an independent study (GSE148729), three cell lines—H1299 lung squamous, Caco-2 colorectal, and Calu-3 lung adenocarcinoma—were infected with SARS-CoV-1 or SARS-CoV-2 and transcriptionally profiled. The SARS-CoV-2 signatures from part c were examined in this additional dataset. Patterns of manifestation of SARS-CoV-2 signatures within the Calu-3 signatures of the independent dataset are highlighted. p values by t test using log2-transformed data. See also Supplementary Data S1.

Figure 1 provides an overview of our study’s approach, utilizing transcriptional gene signatures of the host response to SARS-CoV-2. Independent biological triplicates of three lung cell lines—normal human bronchial epithelium (NHBE), A549 adenocarcinoma, and Calu-3 adenocarcinoma—were mock-treated or infected with SARS-CoV-2 and then profiled for gene expression using RNA-seq^7^. We observed widespread expression changes 24 h post-infection for each cell line, though with notable fewer changes for NHBE than for the adenocarcinoma lines (Supplementary Data S1). At a nominal p value of p < 0.01 (t test), 566 human genes were altered for NHBE (with predicted false discovery rate, or FDR, of 29%), 5675 genes were altered for A549 (FDR 3%), and 4968 genes were altered Calu-3 (FDR 4%). Taking the above genes, supervised clustering of the differential patterns could identify host responses to infection common across multiple cell lines or specific to a single cell line (Fig. 2a). Using a relaxed cutoff for NHBE (one-sided p < 0.05), when combining results with those of A549 and Calu-3, we defined a signature of 308 genes (181 up-regulated, 127 down-regulated) commonly altered in the same direction across all three cell lines (Fig. 2b). We used these data to define four distinct SARS-CoV-2 transcriptional signatures (Fig. 2c): altered in all three cell lines examined (308 genes), altered specifically in A549 (1326 genes), altered specifically in Calu-3 (1327 genes), and altered in the same direction specifically in both A549 and Calu-3 (2963 genes).

Although the A549 cell line has low expression of viral receptor ACE2^7^, the above SARS-CoV-2 signatures shared across multiple cell lines (i.e., NHBE/A549/Calu-3 and A549/Calu-3) were repeatedly observable in A549 cells transduced with ACE2 and infected with SARS-CoV-2 (Supplementary Figure S1). However, the Calu-3-specific transcriptional signature, but not the above A549-specific signature, was also manifested in A549 over-expressing ACE2 (Supplementary Figure S1). The transcriptional differences between A549 with and without ACE2 would presumably have something to do with ACE2 receptor, although NHBE primary cells also expressed ACE2 (Supplementary Figure S1), and so other factors involving the Calu-3 and A549 cancer cell lines, as well as very high ACE2 expression, may also be involved. Notably, alternative receptors with a potential role in SARS-CoV-2 entry may also exist^17^.

In several analyses presented below, we used the above transcriptional signatures of cell line response to SARS-CoV-2 infection as a frame of reference for comparisons with other transcriptional datasets from independent studies, representing other model systems and other diseases. For example, in the above-noted SARS-Cov-2 study, NHBE or A549 cells were infected with other viruses (Influenza A virus or IAV, respiratory syncytial virus or RSV, human parainfluenza virus 3 or HPIV3) or treated with interferon beta (IFNB) and transcriptionally profiled^7^. Across this extended dataset, we examined the differential SARS-CoV-2-associated expression patterns common to all three cell lines (NHBE, A549, Calu-3) or found for just one or two cell lines (Fig. 2c). We observed manifestation of the SARS-CoV-2 signatures within the differential patterns of the other infections and treatments. In this manifestation pattern, the genes up-regulated by SARS-CoV-2 tended to be up-regulated as a group across the other treatments, while the genes down-regulated by SARS-CoV-2 tended to be down-regulated across the other treatments. For example, of the 181 genes up-regulated by SARS-CoV-2 across all three cell lines, 105 were also up-regulated (p < 0.05, t test) in A549 in response to RSV infection. These manifestation patterns were striking when visualized using expression heat maps (Fig. 2c). The manifestation patterns were also evident by Gene Set Enrichment Analysis (GSEA) method (Table S1).

In an independent study (GSE148729), three cell lines—H1299 lung squamous, Caco-2 colorectal, and Calu-3 lung adenocarcinoma—were infected with SARS-CoV-1 or SARS-CoV-2 and transcriptionally profiled. The Calu-3 SARS-CoV-2 signature from the previous dataset validated in the Calu-3 SARS-CoV-1 and SARS-CoV-2 profiles in this additional dataset (Fig. 2d). Of the 181 genes up-regulated by SARS-CoV-2 across all three cell lines from the first dataset, 118 (61%) were also up-regulated (p < 0.05, t test) in SARS-CoV-2-infected Calu-3 in the second dataset. Of the 784 genes up-regulated in the SARS-CoV-2 Calu-3-specific signature (from Fig. 2a), 576 (78%) were also up-regulated in the second Calu-3 dataset. These manifestation patterns were also evident by GSEA method (Table S1). At the same time, differential patterns from the lung squamous and colorectal cell lines did not overlap with those of the previous dataset from normal human bronchial epithelium and lung adenocarcinoma. This lack of signature manifestation may be due to cell type-specific responses, though Calu-3 also had higher *ACE2* expression than either H1299 or Caco-2.

### Pathways associated with the host response to SARS-CoV-2 infection include interferon signaling and inflammation

**Figure 3:**
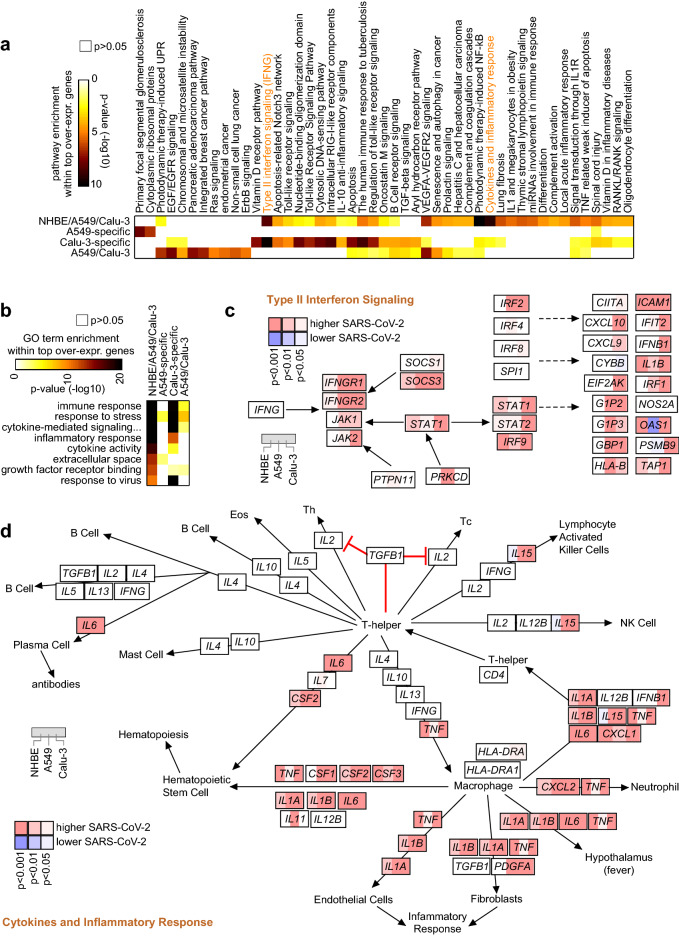
Pathways associated with SARS-CoV-2 infection in lung cells in vitro. (**a**) Significance of enrichment (by one-sided Fisher’s exact test) for wikiPathway^18^ gene sets within the respective sets of genes up-regulated with SARS-CoV-2 infection according to cell line (from Fig. 2b,c). Signatures represented are: up-regulated in all three cell lines examined (NHBE, A549, and Calu-3), A549-specific up-regulation, Calu-3-specific up-regulation, and up-regulated specifically in both A549 and Calu-3. Pathways represented were significant (FDR < 1%^39^) within at least one of the above gene sets. (**b**) Selected significantly enriched Gene Ontology (GO) terms, involving the gene sets described in part a. Enrichment p values by one-sided Fisher’s exact test. (**c**) Pathway diagram representing type II interferon signaling (from wikiPathways), with differential expression patterns in response to SARS-CoV-2 infection in each of the three cell lines represented (left, NHBE; middle, A549; right, Calu-3). Red, high in SARS-CoV-2-infected group versus control. p values by t test using log-transformed values. (**d**) Similar to part c, but for wikiPathway “Cytokines and inflammatory response”. See also Supplementary Data S2 and Supplementary Data S3 (which files provide the numbers of genes involved with each pathway or annotation term).

Each of the four SARS-CoV-2 signatures (three cell lines, A549-specific, Calu-3-specific, and A549/Calu-3) represented specific altered pathways or functional gene categories. We searched wikiPathways^18^ for enrichment of any of our SARS-CoV-2-associated gene sets (Supplementary Data S2). Out of 417 pathways considered, 47 were significant by one-sided Fisher’s exact test with FDR < 1% for at least one of our gene sets (Fig. 3a). Most of these enriched pathways involved the common three cell line signature, with the majority of these pathways also showing enrichment within the Calu-3-specific signature. Enriched pathways within the three cell line signature included “Photodynamic therapy-induced NF-κB survival signaling”, “Cytokines and inflammatory response”, “Type II interferon signaling”, and “VEGFA-VEGFR2 Signaling Pathway”. In terms of functional gene categories, significantly enriched Gene Ontology (GO) annotation terms for the three cell line SARS-CoV-2 signature (Fig. 3b; Supplementary Data S3) included “immune response” (involving 44 out of a total of 741 genes with this annotation), “inflammatory response” (29 out of 370), “cytokine activity” (19 out of 176), “growth factor receptor binding” (13 out of 117), and “response to virus” (15 out of 229). A survey of the wikiPathways “Type II interferon signaling” pathway showed many genes that were statistically significant or trending with the NHBE cell line—including *IFNGR1* and *IFNGR2*, with additional pathway genes significant for A549 or Calu-3 (Fig. 3c). Interestingly, there was little overlap between the Type I and Type II interferon wikiPathways (Supplementary Data S2), with only the latter showing high enrichment for genes up-regulated with SARS-CoV-2 infection. Genes up-regulated with SARS-CoV-2 involving the “Cytokines and Inflammatory Response” pathway (Fig. 3d) included *CSF2*, *CSF3*, *CXCL1*, *CXCL2*, *IL1A*, *IL1B*, and *IL6*.

### The SARS-CoV-2 in vitro transcriptional signatures are manifested in upper airway samples from COVID-19 patients

**Figure 4:**
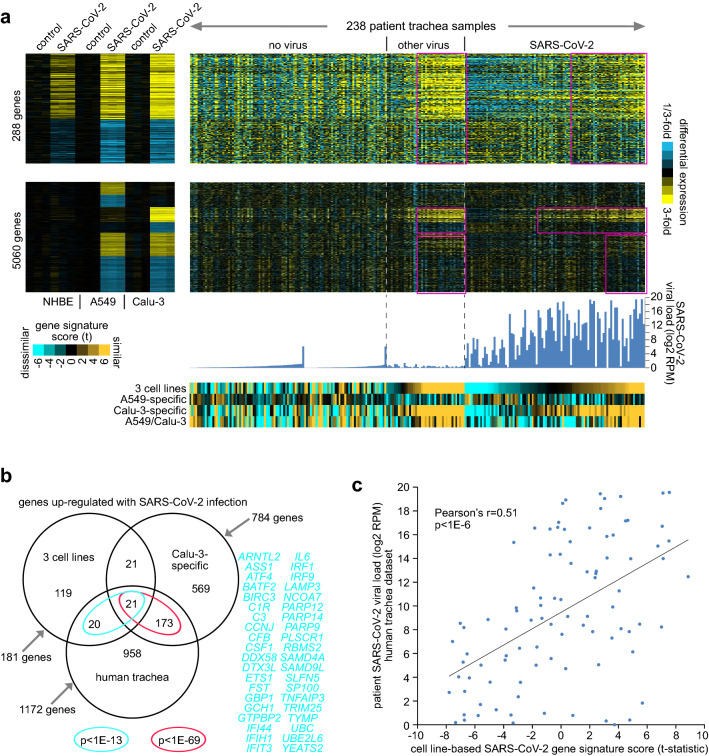
The SARS-CoV-2 in vitro transcriptional signatures are manifested in upper airway samples from COVID-19 patients. (**a**) An RNA-seq dataset of upper airway (trachea) samples in 238 patients with COVID-19, other viral, or non-viral acute respiratory illnesses was examined^8^. The patient expression profiles were probed according to the SARS-CoV-2 transcriptional signatures. Differential SARS-CoV-2-associated expression patterns common to all three cell lines (NHBE, A549, Calu-3) or found for just one or two cell lines (from Fig. 2b,c) are shown for both the in vitro SARS-CoV-2 infection dataset and the patient dataset. Gene order is the same across both datasets. SARS-CoV-2 viral loads (log2 reads per million) corresponding to the patients are also plotted. Heat map contrast (bright yellow/blue) is threefold change from either mock control for the in vitro dataset or from no virus group for the patient dataset. Selected patterns of manifestation of the in vitro SARS-CoV-2 signatures within the human dataset are highlighted. Under the differential expression heat maps, scores for each of the in vitro SARS-CoV-2 signatures across the patient profiles are represented (orange-cyan heatmap). (**b**) Venn diagram representing the gene set overlaps among the genes high with SARS-CoV-2 infection in vitro in all three cell lines (from Fig. 2b), the genes high specifically in Calu-3 lung cell line (from Fig. 2c), and the genes positively correlated with SARS-CoV-2 viral load across the 238 patient samples (p < 0.01, Pearson’s correlation using log2-transformed data). p values by one-sided Fisher’s exact test. Genes overlapping between three cell line gene set and patient gene set are listed. (**c**) Scatterplot of cell line-based SARS-CoV-2 gene signature score versus SARS-CoV-2 viral load, for the 94 COVID-19 patients in the human trachea dataset. See also Supplementary Data S4.

To determine the relevance of our SARS-CoV-2 in vitro signatures to clinical samples, we examined an RNA-seq dataset of upper airway (trachea) samples in 238 patients with COVID-19, other viral, or non-viral acute respiratory illnesses^8^. We scored each patient sample expression profile for each of the four SARS-CoV-2 transcriptional signatures (three cell lines, A549-specific, Calu-3-specific, and A549/Calu-3). The scoring basis was on whether the relative differential patterns in the patient sample profile—higher versus lower—were broadly similar to the patterns of up- or down-regulation, respectively, in the in vitro infection dataset. As compared to the non-viral group, SARS-CoV-2 in vitro scores for the common three cell line signature and the Calu-3-associated signatures were higher in a substantial fraction of patient samples in both the COVID-19 and other viral groups (Fig. 4a). Taking the top set of genes correlated positively with SARS-CoV-2 viral load across the 238 patient samples (p < 0.01, Pearson’s correlation), these significantly overlapped with the genes high in the in vitro three cell line signature (41 genes out of 181, p < 1E−13, one-sided Fisher’s exact test) and with the genes high in the Calu-3-specific signature (194 out of 784 genes, p < 1E−69, Fig. 4b; Table S1). The overlapping genes involved pathways related to cytokines and the inflammatory response. Samples from COVID-19 patients with high viral loads of SARS-CoV-2 tended to most strongly manifest the in vitro signatures of infection, while samples from lower viral loads often appeared negative for the signatures (Fig. 4a,c; Supplementary Data S4).

### The SARS-CoV-2 transcriptional signatures represent broad similarities with host responses to other viruses

**Figure 5:**
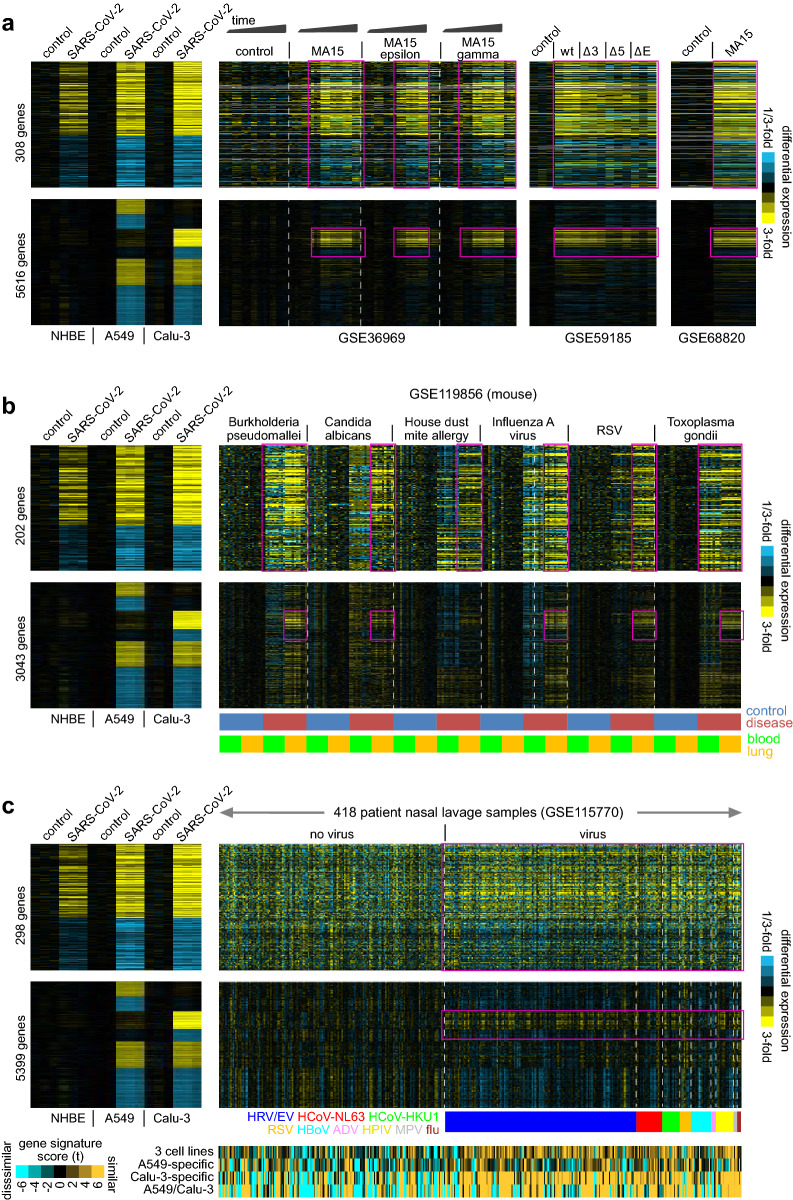
The SARS-CoV-2 in vitro transcriptional signatures overlap with signatures of host responses to other virus in other model systems and patient samples. (**a**) Gene expression profiles of lung samples from mice infected with SARS viruses other than SARS-CoV-2 were probed according to the SARS-CoV-2 transcriptional signatures. Differential SARS-CoV-2-associated expression patterns common to all three cell lines (NHBE, A549, Calu-3) or found for just one or two cell lines (from Fig. 2b,c) are shown for both the in vitro SARS-CoV-2 infection dataset and the three independent SARS mouse datasets (GSE36969, GSE59185^19^, GSE68820^20^). Gene order is the same across all datasets. Mouse datasets involve different variants of the SARS-CoV-MA15 virus, in addition to wild-type. (**b**) A gene expression profiling dataset of blood and lung samples obtained from mice infected or challenged with Toxoplasma gondii, Influenza A virus, Respiratory Syncytial virus (RSV), acute Burkholderia pseudomallei, Candida albicans, and House dust mite (HDM) allergen (GSE119856^21^) was probed for the SARS-CoV-2 transcriptional signatures. Gene order is the same for both datasets. Selected patterns of manifestation of SARS-CoV-2 signatures within the GSE119856 mouse lung samples are highlighted. (**c**) A gene expression dataset of 418 patient from both viral-associated and non-viral nasal lavage samples^22^ (GSE115770) was probed for the SARS-CoV-2 transcriptional signatures. Gene order is the same for both datasets. GSE115770 profiles are ordered by virus (HRV/EV, HCoV-NL63, HCoV-HKU1, RSV, HBoV, ADV, HPIV, MPV, flu). Under the differential expression heat maps, scores for each of the SARS-CoV-2 signatures across the GSE115770 profiles are represented (orange-cyan heatmap).

Extending upon the analysis results from the GSE147507 dataset (Fig. 2c), we examined transcriptional data from other viruses in other model systems, including mice, to compare the host response of SARS-CoV-2 with the host responses of other viruses (Supplementary Data S1). The common three cell line signature and the Calu-3 specific signature of SARS-CoV-2 infection were broadly discernable in transcriptional profiles of lung samples from mice infected with other SARS coronaviruses (Fig. 5a; Table S1), based on analysis of three external public datasets (GSE36969, GSE59185^19^, GSE68820^20^). Of the 308 genes in our SARS-CoV-2 three cell line signature (from Fig. 2a), 181 (59%) were significantly altered in the same direction (p < 0.05) for at least one of the three SARS datasets in mice. Similarly, in another gene expression profiling dataset of blood and lung samples in mice, the three cell line signature and the Calu-3 specific signature of SARS-CoV-2 infection shared broad similarities with host responses to Toxoplasma gondii, Influenza A virus, RSV, acute Burkholderia pseudomallei, Candida albicans, and House dust mite^21^ (Fig. 5b; Table S1). In the above dataset, we found more commonalities with the SARS-CoV-2 signatures for the mouse lung samples than for the blood samples.

We next examined human samples, using a gene expression dataset of 418 patients from both viral-associated and non-viral nasal lavage samples^22^, with viruses represented including Human Rhino/enteroviruses (HRV/EV), Human coronavirus NL63 (HCoV-NL63), Human coronavirus HKU1 (HCoV-HKU1), RSV, Human bocavirus (HBoV), Aleution disease virus (ADV), human parainfluenza virus (HPIV), Human metapneumovirus (MPV), and influenza. The SARS-CoV-2 signatures were again broadly similar to the signatures of other viruses (Fig. 5c; Table S1), and the scores for each SARS-CoV-2 signature were significantly higher in the viral group compared to the non-viral group. In particular, the three cell line and Calu-3 signatures appeared markedly elevated in the viral group (p < 1E−11, t test).

### The SARS-CoV-2 transcriptional signatures are manifested in a subset of human lung tumors involving the immune checkpoint pathway

As the A549 and Calu-3 lung adenocarcinoma cell lines each exhibited distinctive transcriptional signatures of SARS-CoV-2 infection, we sought to determine whether they might be manifested within a subset of human lung tumors. We examined the SARS-CoV-2 transcriptional signatures in The Cancer Genome Atlas (TCGA) Non-Small Cell Lung Cancer (NSCLC) cohort of 1016 cases (primarily adenocarcinomas and squamous cell carcinomas)^16^. Using the mRNA profiling dataset from TCGA, we scored each tumor expression profile for each of the four SARS-CoV-2 signatures (three cell lines, A549-specific, Calu-3-specific, and A549/Calu-3). A previous study classified the NSCLC profiles into nine molecular subtypes^16^, three associated with lung squamous cell carcinoma, and six associated with lung adenocarcinoma. Three of the adenocarcinoma subtypes—AD.2, AD.3, and AD.4—express several immune checkpoint genes, including PDL1 and PDL2, corresponding with patterns of greater immune cell infiltration^16^.

**Figure 6:**
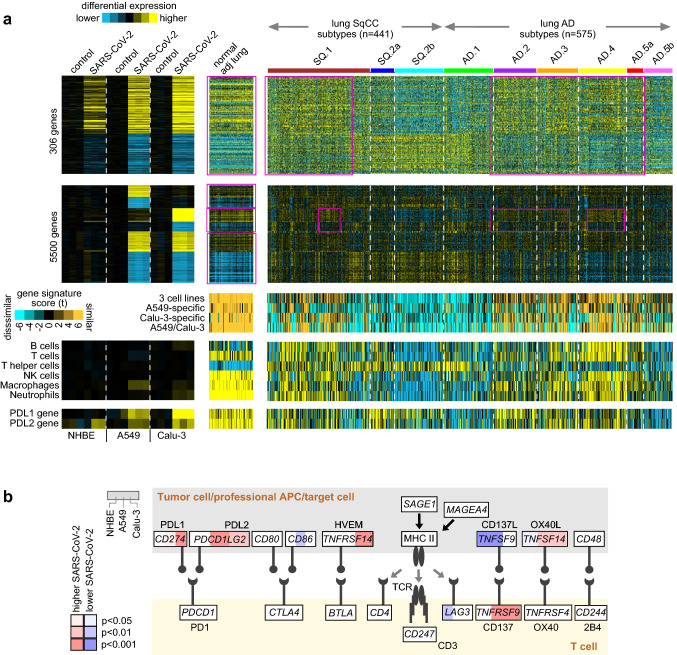
The SARS-CoV-2 transcriptional signatures are manifested in subsets of human lung cancer involving the immune checkpoint pathway. (**a**) RNA-seq profiles of 1023 Non-Small Cell Lung Cancer (NSCLC) cases^16^ were probed according to the SARS-CoV-2 transcriptional signatures. Differential SARS-CoV-2-associated expression patterns common to all three cell lines (NHBE, A549, Calu-3) or found for just one or two cell lines (from Fig. 2b,c) are shown for both the in vitro SARS-CoV-2 infection dataset and the NSCLC dataset (“normal adj lung”, normal adjacent lung tissue samples in proximity to lung tumor, n = 110). Gene order is the same across both datasets. The ordering of NSCLC profiles is by nine previously-identified molecular subtypes^16^, three associated with lung squamous cell carcinoma (SqCC) and six associated with lung adenocarcinoma (AD). Heat map contrast (bright yellow/blue) is threefold change from control for SARS-CoV-2 dataset and 1 SD from median for NSCLC dataset. Selected patterns of manifestation of SARS-CoV-2 signatures within the human cancers are highlighted. Under the differential expression heat maps, scores for each SARS-CoV-2 signature across the NSCLC profiles are represented (orange-cyan heatmap). Gene expression-based signatures of immune cell infiltrates^38^ and PDL1/PDL2 genes are also represented in both SARS-CoV-2 and NSCLC datasets (NK cells, natural killer cells). (**b**) Diagram of immune checkpoint pathway (featuring interactions between T cells and antigen-presenting cells, including tumor cells), with differential expression patterns in response to SARS-CoV-2 infection in each of the three cell lines represented (left, NHBE; middle, A549; right, Calu-3). Red, high in SARS-CoV-2-infected group versus control. p values by t test using log-transformed values. Most all of the genes represented where previously found elevated across the AD.2, AD.3, and AD.4 NSCLC subtypes^16^.

We found that the common signature of SARS-CoV-2 infection across three cell lines represented a transcriptional program associated with the immune response and immune checkpoint pathway in human lung tumors. Interestingly, scores for all four SARS-CoV-2 signatures had higher levels in normal adjacent lung tissues than in lung tumors (Fig. 6a, Table S2, Supplementary Data S4). Normal adjacent tissues involve inflammation and immune cell infiltration, as well as a collection of cell types that differ from the cancer cell of origin. The common three cell line signature, in the TCGA lung tumor profiles, was uniformly manifested across the SQ.1, AD.2, AD.3, and AD.4 subtypes in particular (Table S2). Scores for the other three signatures related to A549 or Calu-3 were broadly correlated with those of the three cell line signature, though with less distinctive associations according to NSCLC subtype. As expected, the SARS-CoV-2-infected cell lines did not show signatures of immune cell infiltrates found in the above lung tumor subtypes, as tumors represent a mixture of cancer and non-cancer cells in contrast to cell lines. However, both A549 and Calu-3 showed elevated expression (p < 0.05, t test using log2-transformed data) of immune checkpoint genes *CD274* (PDL1) and *PDCD1LG2* (PDL2) with SARS-CoV-2 infection (Fig. 6a,b). A survey of immune checkpoint pathway genes^16^ also showed up-regulation of *TNFSF14* in A549 and Calu-3 in response to infection and *TNFRSF14* in Calu-3. Previously, most immune checkpoint-related genes, those presumed to express in either T-cells or the target cells, have been found elevated across the AD.2, AD.3, and AD.4 NSCLC subtypes^16^.

### The SARS-CoV-2 transcriptional signatures are manifested across large subsets of human cancers from diverse histopathological types

We sought to determine the relevance of our SARS-CoV-2 signatures to cancer types other than lung. We hypothesized that the transcriptional programs associated with viral infection in vitro could be manifested within well-defined subsets of human tumors. Therefore, we examined the entire TCGA pan-cancer cohort of 10,224 cases involving 32 major types and previously classified into ten major pan-cancer classes that cut across the tissue of origin^14^. These ten pan-cancer classes included a “c3” class (representing ~ 13% of all cancers), strongly associated with the immune response and immune checkpoint pathways, and “c7” and “c8” classes (representing ~ 11% and 9% of cancers, respectively), associated with mesenchymal or stromal cells. The c3, c7, and c8 classes also associated with hypoxia, NRF2/KEAP1, Wnt, and Notch pathways^14^. Using the mRNA profiling dataset from TCGA, we scored each tumor expression profile for each of the four SARS-CoV-2 signatures (three cell lines, A549-specific, Calu-3-specific, and A549/Calu-3).

**Figure 7:**
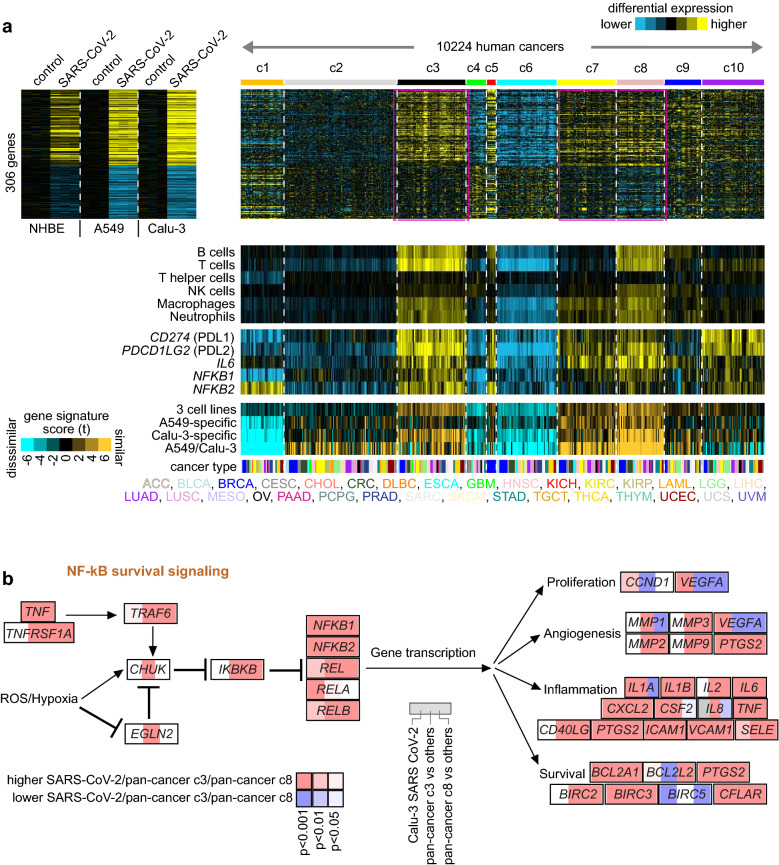
The SARS-CoV-2 transcriptional signatures are manifested in specific pan-cancer classes involving multiple tissues of origin, the immune response, and NF-κB signaling. (**a**) RNA-seq profiles of 10,224 cancer cases across 32 major types were previously classified into ten molecular-based pan-cancer “classes” (profiles normalized within their respective cancer types)^14^. Differential SARS-CoV-2-associated expression patterns common to all three cell lines (NHBE, A549, Calu-3) or found for just one or two cell lines (from Fig. 2b,c) are shown for both the in vitro SARS-CoV-2 infection dataset and the pan-cancer dataset. Gene order is the same across both datasets. Human tumor profiles are ordered by pan-cancer class, with cancer type based on tissue of origin and histopathology indicated along the bottom. Heat map contrast (bright yellow/blue) is threefold change from control for SARS-CoV-2 dataset and 1 SD from median for pan-cancer dataset. Selected patterns of manifestation of SARS-CoV-2 signatures within the human cancers are highlighted, involving c3 class (immune-related) and c7 and c8 classes (mesenchymal- or stroma-related). Under the differential expression heat maps, scores for each SARS-CoV-2 signature across the human tumor profiles are represented (orange-cyan heatmap). Gene expression-based signatures of immune cell infiltrates^38^ and selected genes are also represented across the human tumor profiles (NK cells, natural killer cells). (**b**) Diagram of the NF-κB signaling pathway (from wikiPathways^18^, full pathway name “Photodynamic therapy-induced NF-κB survival signaling”). For each gene shown, three different expression comparisons are represented: SARS-CoV-2 infection versus control in Calu-3 cells (left square), pan-cancer c3 class versus the other tumors (middle square), and pan-cancer c8 class versus the other tumors (right square). Red, high expression in SARS-CoV-2-infected cells, c3 human tumors, or c8 human tumors, as indicated. p values by t test using log-transformed values. See also Supplementary Data S5.

We found that both the three cell line and Calu-3-specific signatures associated with SARS-CoV-2 infection manifested in the c3, c7, and c8 human tumors, though more prominently in c3 and c8 (Fig. 7a, Table S2, Supplementary Data S4, S5). Of the 181 genes up-regulated by SARS-CoV-2 across all three cell lines, 150 (83%) were also up-regulated (p < 0.01, t test) in c3 compared to other human tumors, and 111 (61%) were also up-regulated (p < 0.01) in c8 compared to other tumors (Supplementary Data S5). The SARS-CoV-2 signature specific to A549 and Calu-3 but not NHBE also manifested in c7 and c8 tumors. The set of genes both high in the three cell line SARS-CoV-2 signature and high in either c3 or c8 human tumors versus other tumors (p < 0.01, t test) were enriched for a similar set of wikiPathways associated above with SARS-CoV-2 alone (Fig. 3a; Supplementary Data S5). Enriched pathways common to SARS-CoV-2 infection and c3 and c8 human tumors included the NF-κB survival signaling pathway, including *NFKB1*, *NFKB2*, *REL*, and *RELB* genes, as well as downstream transcriptional targets (Fig. 7b). These findings were specific, as we found that other pathways previously associated with c3 and c8 (e.g., Wnt and Notch) were not associated with SARS-CoV-2 infection.

## Discussion

In our present study, we have shown that the host transcriptional response to SARS-CoV-2 infection, as identified using cell lines, shares broad similarities with results from multiple independent studies of coronaviruses or other viruses, using other model systems or patient samples. Our results demonstrate how in vitro model system could be effective in identifying rapid responses within cancer cells that would also be observable in other cellular contexts. In particular, the Calu-3 model showed specific host responses to infection not observed in the other two cell lines but which validated in independent datasets. In particular, the overall similarities in host responses observed among different coronaviruses support some degree of leveraging of what has previously been learned towards our understanding of SARS-CoV-2. In the United States, current guidelines from the Centers for Disease Control (CDC) require Biosafety Level (BSL)-3 facilities and practices for experimental studies involving the SARS-CoV-2 virus, which can be rather restrictive in practice. For some proposed studies, coronaviruses with lower Biosafety Levels, such as 229E or NL63, might yield similar results to those of SARS-CoV-2.

The meta-analysis results across the various datasets, as provided in our supplemental, represents a resource for future investigations, whereby one can identify gene candidates for the study of the host response that appear common to multiple systems or viruses. Our data could also help identify genes that would be specific to coronaviruses or SARS-CoV-2 in particular. Genes that appear involved with COVID-19 in both experimental models and human patient samples may be particularly attractive for further study, and our results allow for honing in on a focused set of genes. However, identifying genes altered specifically in response to SARS-CoV-2 infection and not in response to any other viral infection may be challenging, as asserting a negative is inherently difficult. Our results also indicate that different cell types may respond differently to viral infection. For example, host response patterns observed in lung adenocarcinoma cells did not show in colon or lung squamous cells.

Our study further revealed that the transcriptional programs initiated in cancer cells in response to SARS-CoV-2 are also at work within ~ 20% of human tumors. This finding reflects known parallels between responses to viral infection and the immune response associated with cancer^10^. These associations would not be exclusive to SARS-CoV-2 but would involve host responses to a broad range of viruses. The viral response signatures manifest within specific and previously-identified cancer subtypes, which strongly indicates that the signatures represent a coordinated transcriptional program that underlies these subtypes. The inherent limitations of cancer cell line models, e.g., in their inability to capture the microenvironmental effects at work within human tumors, are well understood. At the same time, cell lines can reveal molecular properties intrinsic to cancer cells, independent of cellular environment or context. Tumors represent a mixture of cancer and non-cancer cells, which may include immune cells, and distinguishing between the two based on molecular data on bulk tumor samples is inherently difficult. In contrast, the cell line results allow us to de-convolute an immune response program that would be activated specifically within the cancer cells of the tumor. The in vitro viral infection model could help identify candidate genes with roles in cancer cell responses to immune cells and inflammation. For example, we found cancer-specific responses to viral infection to include up-regulation of PDL1 and PDL2 genes, two critical targets in cancer immunotherapy^23^. As cancer represents a collection of molecularly heterogeneous diseases, different cell lines may respond differently to infection, as observed here.

The links identified here between viral infection and cancer suggest opportunities for leveraging knowledge between domains^10^. Pathways identified as part of the host response to viral infection could be relevant for therapeutic targeting, both for certain viral infections and specific cancer subsets. Inflammation and immunity are inherent characteristics of cancer, and both viruses and cancers are associated with dominant Th1 responses^10^, as reflected in our results. The host response to SARS-CoV-2 includes interleukin-6 (IL-6)^7^, which plays an important role in the “cytokine storm,” and IL-6 receptor antagonist tocilizumab is currently under evaluation as a treatment for severe COVID-19^24^. IL-6 is a major factor driving T helper 17 (Th17) responses^25^, which, under some circumstances, can interfere with the control of viral infections. Similarly, Th17 responses can either promote or inhibit tumorigenesis, depending on the precise tumor and other factors^26,27^. IL-6 also promotes tumorigenesis by regulating multiple hallmarks of cancer and signaling pathways. As such, blocking IL-6 is under investigation as an anticancer therapy^28^, but our findings suggest that strategies that block Th17 responses might offer additional benefit in the context of COVID-19. Similarly, NF-κB-mediated inflammation, known to be associated with several cancer types^29^, has also been investigated previously as a therapeutic target for SARS coronaviruses^30^, which findings would likely apply to SARS-CoV-2. Our present study has brought together disparate results from multiple systems, diseases, and domains. These results can lend support to current therapeutic strategies under investigation, as well as suggest new ones.

## Materials and methods

### Derivation of SARS-CoV-2 transcriptional signatures of host response in cell lines

To define transcriptional signatures of the host cell response to SARS-CoV-2 infection, we referred to the GSE147507 dataset^7^. In this dataset, three lung cell lines—NHBE, A549, and Calu-3—were mock-treated or infected with SARS-CoV-2 and then profiled for gene expression using RNA-seq^7^. We used data from the SARS-CoV-2 profiling experiments involving multiplicity-of-Infection (MOI) of 2. We converted raw gene-level sequencing read counts to reads per million Mapped (RPM) values and then log2-transformed them.

Using GSE147507, we defined a common set of 308 genes up-regulated or down-regulated across all three cell lines and in the same direction of change (one-sided p < 0.05 NHBE, and two-sided p < 0.01 A549 and Calu-3, t test using log2-transformed expression). For NHBE, we used a relaxed statistical cutoff, to lower false negatives, as we combined the NHBE results with results from A549 and Calu-3. A gene in the common three cell line signature had to meet multiple criteria for inclusion, which mitigated the relatively high FDR (adjusted for multiple testing) observed when considering NHBE alone. Also, significant patterns of correspondence were observed when examining the common signature across multiple independent datasets.

In addition, we evaluated the set of genes differentially expressed for any one of the three cell lines, with p < 0.01 (t test using log2-transformed data) in infection versus mock-treated, to identify patterns specific to one cell line or common across multiple cell lines. We performed this supervised clustering approach^31^ as follows: (1) expression values within each cell line were centered on the average of the corresponding control group; (2) each pattern of interest (i.e., genes up-regulated or down-regulated specifically in A549, or genes up-regulated or down-regulated in both A549 and Calu-3 but not NHBE) was represented as a series of 1 s and 0 s; (3) for each gene, we computed the Pearson’s correlation between its expression values and each of the predefined patterns; (4) for each genes, the pre-defined pattern of interest best correlated with the gene’s differential expression pattern was determined; and (5) we sorted the genes by their assigned patterns. The dominant signatures from this analysis included an A549-specific signature, a Calu-3-specific signature, and an A549/Calu-3 common signature. A subset of genes in the common three cell line signature was also part of the cell line-specific signatures, representing instances where the gene was differentially expressed in all three cell lines, but with the altered expressed being particularly prominent within one or two cell lines. Of the 308 genes in the three cell line signature, 47 were included in the Calu-3-specific signature, and 165 were included in the A549/Calu-3 common signature.

### Pathway analyses

We searched each of the four SARS-CoV-2 in vitro signatures for enrichment of previously-curated pathways and functional gene groups. We evaluated enrichment of GO annotation terms^32^ and wikiPathways^18^ within sets of genes up-regulated in response to viral infection, using SigTerms software^33^ and one-sided Fisher’s exact tests. Gene sets for each wikiPathway were downloaded in July 2019 (“20190710” version). For GO term enrichment analysis, we used all 19510 unique proteins represented in at least one of the seven cancer types profiled as the reference population. For wikiPathways enrichment analysis, we used all 6597 unique proteins represented in at least one wikiPathway as the reference population.

### Analysis of external transcriptome datasets

For multiple viral and human cancer datasets, we scored each external mRNA profile according to the in vitro SARS-CoV-2 transcriptional signatures from GSE147507 dataset (three cell lines, A549-specific, Calu-3-specific, and A549/Calu-3). The scoring basis was on whether the relative differential patterns in the external sample profile, higher versus lower, were broadly similar to the patterns of up- versus down-regulation, respectively, in the in vitro SARS-CoV-2 signature. We based the SARS-CoV-2 signature score on our previously described “t score” metric^34–37^. We have defined the t score as the two-sided t statistic when comparing, within each external differential expression profile, the average of the SARS-CoV-2-up-regulated genes with the average of the down-regulated genes. For example, the t score for a given sample profile is high when both the up-regulated genes in the signature are high and the down-regulated genes are low. For viral expression datasets, we centered logged expression values (base 2) on the corresponding non-viral group. For TCGA lung^16^, and pan-cancer^14^ datasets, logged expression values for each gene were centered on the median and divided by the standard deviation across the sample profiles. For TCGA pan-cancer dataset^14^, logged expression values for each gene were centered on the median and divided by the standard deviation within their respective cancer types (according to TCGA project). Computational inference of the infiltration levels of specific immune cell types using RNA-seq data, based on published immune signatures^38^, was carried out previously for TCGA datasets^14,16^. RPM values for the COVID-19 patient trachea dataset^8^ were quantile normalized before the analysis.

When joining genes from microarray datasets to the GSE146507 dataset, for side-by-side comparisons of the differential patterns using heat maps, there were cases where multiple array probes referred to the same gene. In these cases, we used the probe with either the smallest p value (in either direction, where the dataset involved just two experimental groups) or the highest standard deviation across sample profiles (where multiple experimental groups were involved) to represent the gene.

### Statistical analysis

All p values were two-sided unless otherwise specified. We performed all tests using log2-transformed gene expression values. False Discovery Rates (FDRs) were estimated using the method of Storey and Tibshirini^39^. Visualization using heat maps was performed using both JavaTreeview (version 1.1.6r4)^40^ and matrix2png (version 1.2.1)^41^. GSEA^42^ was carried out using version 4.0.3 of the software, using weighted enrichment statistic and 10,000 gene set permutations. For GSEA, genes were ranked using GSEA’s Signal2Noise metric, except for human trachea COVID-19 dataset, which used correlation with log2 viral load for the gene rankings.

## Supplementary Information

Supplementary Information 1.

Supplementary Information 2.

Supplementary Information 3.

Supplementary Information 4.

Supplementary Information 5.

Supplementary Information 6.

## Data Availability

All data used in this study are publicly available. We obtained RNA-seq or microarray expression data from experimental models of viral infection or other treatments from the Gene Expression Omnibus (GEO). The COVID-19 trachea patient RNA-seq dataset is available at https://github.com/czbiohub/covid19-transcriptomics-pathogenesis-diagnostics-results and at GEO (GSE156063). TCGA data are available through the Genome Data Commons (https://gdc.cancer.gov/) and the Broad Institute’s Firehose data portal (https://gdac.broadinstitute.org).

## Abbreviations

NHBE: Normal Human Bronchial Epithelium
TCGA: The Cancer Genome Atlas
COVID-19: Coronavirus disease 19
SARS-CoV-2: Severe acute respiratory syndrome coronavirus 2

## References

[1] Dong E, Du H, Gardner L (2020). An interactive web-based dashboard to track COVID-19 in real time. Lancet Infect. Dis..

[2] Nicola M (2020). The socio-economic implications of the coronavirus pandemic (COVID-19): A review. Int. J. Surg..

[3] Gao, Z. *et al.* A systematic review of asymptomatic infections with COVID-19. *J. Microbiol. Immunol. Infect.* **(E-pub May 15)** (2020). 10.1016/j.jmii.2020.05.001 PMC7227597 32425996

[4] Chow N (2020). Preliminary estimates of the prevalence of selected underlying health conditions among patients with coronavirus disease 2019—United States, February 12–March 28, 2020. Morb. Mortal Wkly. Rep..

[5] Hotez P, Bottazzi M, Corry D (2020). The potential role of Th17 immune responses in coronavirus immunopathology and vaccine-induced immune enhancement. Microbes Infect..

[6] Murthy S, Gomersall C, Fowler R (2020). Care for critically ill patients with COVID-19. JAMA.

[7] Blanco-Melo D (2020). Imbalanced host response to SARS-CoV-2 drives development of COVID-19. Cell.

[8] Mick E (2020). Upper airway gene expression reveals suppressed immune responses to SARS-CoV-2 compared with other respiratory viruses. Nat. Commun..

[9] Zapatka M (2020). The landscape of viral associations in human cancers. Nat. Genet..

[10] Goldszmid R, Dzutsev A, Trinchieri G (2014). Host immune response to infection and cancer: Unexpected commonalities. Cell Host Microbe.

[11] Thorsson V (2018). The immune landscape of cancer. Immunity.

[12] Hanahan D, Weinberg R (2011). Hallmarks of cancer: The next generation. Cell.

[13] Hanahan D, Weinberg R (2000). The hallmarks of cancer. Cell.

[14] Chen F (2018). Pan-cancer molecular classes transcending tumor lineage across 32 cancer types, multiple data platforms, and over 10,000 cases. Clin. Cancer Res..

[15] Gonzalez-Cao, M. *et al.* Activation of viral defense signaling in cancer. *Ther. Adv. Med. Oncol.* **(E-pub Aug 29)** (2018). 10.1177/1758835918793105 PMC6116077 30181782

[16] Chen, F. *et al.* Multiplatform-based molecular subtypes of non-small cell lung cancer. *Oncogene* **(E-pub Oct 24)** (2016). 10.1038/onc.2016.303 PMC5344748 27775076

[17] Cuervo N, Grandvaux N (2020). ACE2: Evidence of role as entry receptor for SARS-CoV-2 and implications in comorbidities. Elife.

[18] Slenter D (2018). WikiPathways: A multifaceted pathway database bridging metabolomics to other omics research. Nucleic Acids Res..

[19] Regla-Nava J (2015). Severe acute respiratory syndrome coronaviruses with mutations in the E protein are attenuated and promising vaccine candidates. J. Virol..

[20] Totura A (2015). Toll-like receptor 3 signaling via TRIF contributes to a protective innate immune response to severe acute respiratory syndrome coronavirus infection. mBio.

[21] Singhania A (2019). Transcriptional profiling unveils type I and II interferon networks in blood and tissues across diseases. Nature.

[22] Altman M (2019). Transcriptome networks identify mechanisms of viral and nonviral asthma exacerbations in children. Nat. Immunol...

[23] Chen D, Mellman I (2017). Elements of cancer immunity and the cancer-immune set point. Nature.

[24] Zhang C, Wu Z, Li J-W, Zhao H, Wang G-Q (2020). Cytokine release syndrome in severe COVID-19: Interleukin-6 receptor antagonist tocilizumab may be the key to reduce mortality. Int. J. Antimicrob. Agents.

[25] Kimura A, Kishimoto T (2010). IL-6: Regulator of Treg/Th17 balance. Eur. J. Immunol..

[26] Ye J, Livergood R, Peng G (2013). The role and regulation of human Th17 cells in tumor immunity. Am. J. Pathol..

[27] You R (2018). IL17A regulates tumor latency and metastasis in lung adeno and squamous SQ.2b and AD.1 cancer. Cancer Immunol. Res..

[28] Kumari N, Dwarakanath B, Das A, Bhatt A (2016). Role of interleukin-6 in cancer progression and therapeutic resistance. Tumour Biol..

[29] Pires B, Silva R, Ferreira G, Abdelhay E (2018). NF-kappaB: Two sides of the same coin. Genes (Basel).

[30] DeDiego M (2014). Inhibition of NF-κB-mediated inflammation in severe acute respiratory syndrome coronavirus-infected mice increases survival. J. Virol..

[31] Fernandez-Valdivia, R. *et al.* Transcriptional response of the murine mammary gland to acute progesterone exposure. *Endocrinology* **(E-pub Aug 7)** (2008). 10.1210/en.2008-0768 PMC2613059 18687774

[32] Ashburner M (2000). Gene ontology: Tool for the unification of biology. The Gene Ontology Consortium. Nat. Genet..

[33] Creighton C, Nagaraja A, Hanash S, Matzuk M, Gunaratne P (2008). A bioinformatics tool for linking gene expression profiling results with public databases of microRNA target predictions. RNA.

[34] Cancer Genome Atlas Research Network (2011). Integrated genomic analyses of ovarian carcinoma. Nature.

[35] Creighton C (2012). Integrated analyses of microRNAs demonstrate their widespread influence on gene expression in high-grade serous ovarian carcinoma. PLoS One.

[36] The Cancer Genome Atlas Research Network (2013). Comprehensive molecular characterization of clear cell renal cell carcinoma. Nature.

[37] Creighton C (2009). Residual breast cancers after conventional therapy display mesenchymal as well as tumor-initiating features. Proc. Natl. Acad. Sci. USA.

[38] Bindea G (2013). Spatiotemporal dynamics of intratumoral immune cells reveal the immune landscape in human cancer. Immunity.

[39] Storey JD, Tibshirani R (2003). Statistical significance for genomewide studies. Proc. Natl. Acad. Sci. USA.

[40] Saldanha AJ (2004). Java Treeview–extensible visualization of microarray data. Bioinformatics.

[41] Pavlidis P, Noble W (2003). Matrix2png: A utility for visualizing matrix data. Bioinformatics.

[42] Subramanian A (2005). Gene set enrichment analysis: A knowledge-based approach for interpreting genome-wide expression profiles. Proc. Natl. Acad. Sci. USA.

