# Supplementary material for: Meta-analysis of host transcriptional responses to SARS-CoV-2 infection reveals their manifestation in human tumors
Source: Sci Rep. 2021 Jan 28;11:2459. doi: 10.1038/s41598-021-82221-4 (PMC7844278; doi:10.1038/s41598-021-82221-4)

**Supplementary Figures**

**Meta-analysis of host transcriptional responses to SARS-CoV-2 infection reveals their manifestation in human tumors**

Fengju Chen^1*^, Yiqun Zhang^1*^, Richard Sucgang^2^, Sasirekha Ramani^2^, David Corry^3,4,5,6^, Farrah Kheradmand^3,4,5,6^, Chad J. Creighton^1,6,7,8^

1. Dan L. Duncan Comprehensive Cancer Center Division of Biostatistics, Baylor College of Medicine, Houston, TX 77030, USA.
2. Department of Molecular Virology and Microbiology, Baylor College of Medicine, Houston, TX, 77030, USA.
3. Center for Translational Research in Inflammatory Diseases, Michael E. DeBakey VA, Houston, TX 77030,Texas.
4. Departments of Pathology and Immunology, Baylor College of Medicine, Houston, TX 77030, Texas.
5. Biology of Inflammation Center, Baylor College of Medicine, Houston, TX 77030, Texas.
6. Department of Medicine, Baylor College of Medicine, Houston, TX 77030, USA.
7. Department of Bioinformatics and Computational Biology, The University of Texas MD Anderson Cancer Center, Houston, TX 77030, USA.
8. Human Genome Sequencing Center, Baylor College of Medicine, Houston, TX 77030, USA.


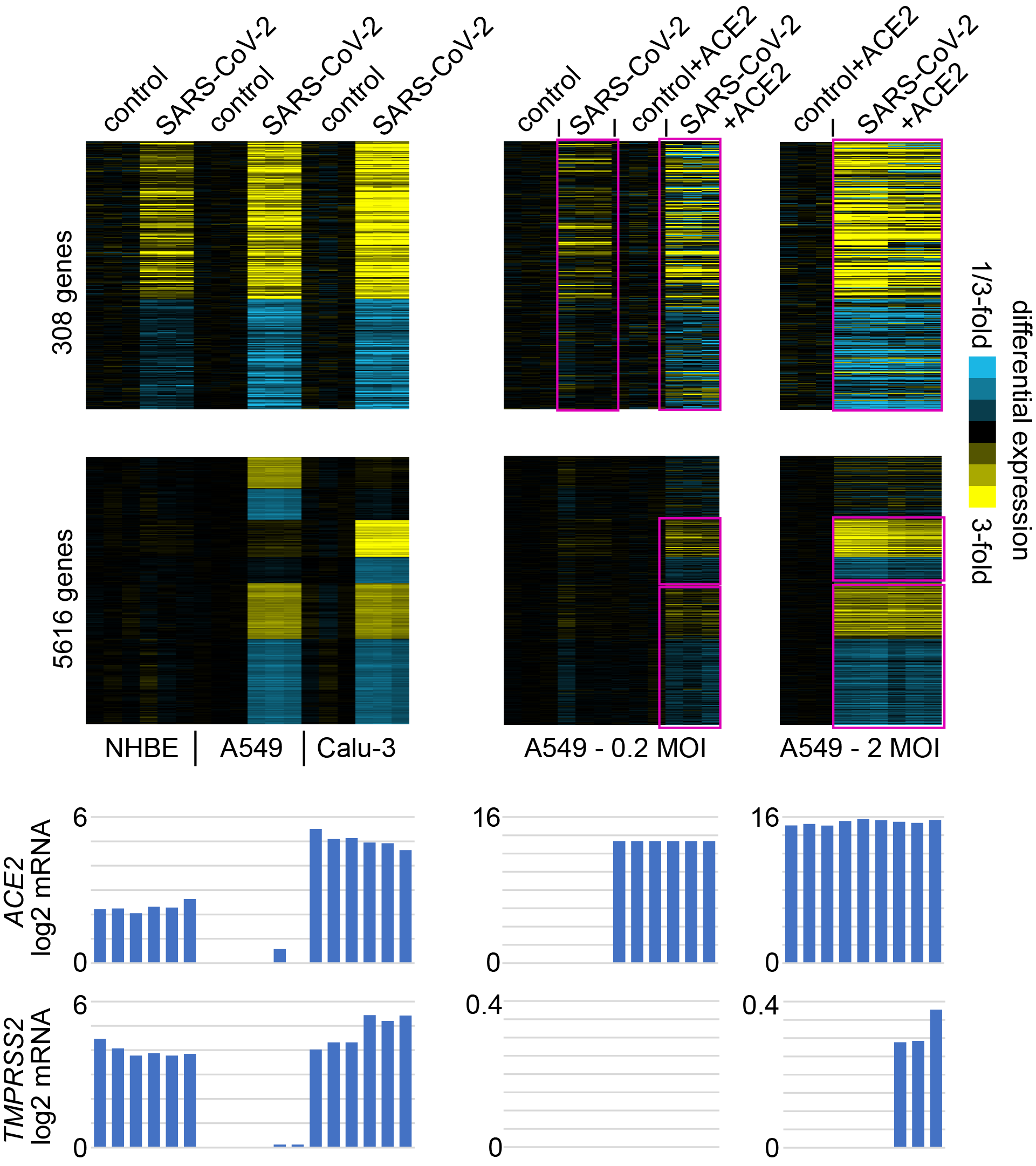


**Supplementary Figure 1. The SARS-CoV-2 transcriptional signatures are manifested in A549 cells expressing ACE2 and infected with SARS-CoV-2.** In the same GSE157407 study used to derive the SARS-CoV-2 transcriptional signatures (Figure 2), A549 cells were infected with SARS-CoV-2 at MOI 0.2 or MOI 2 and transduced with a vector expressing mCherry or ACE2. Differential SARS-CoV-2-associated expression patterns common to all three cell lines (NHBE, A549, Calu-3) or found for just one or two cell lines (taken from Figure 2a) are shown for both the first set of SARS-CoV-2 infection profiles and the additional profiles in A549 representing MOI 0.2 and ACE2 transfection, as indicated. Each treatment profile is centered on the average of its corresponding control group. Patterns of manifestation of SARS-CoV-2 signatures within the other virus or treatment groups are highlighted. Expression levels of *ACE2* and *TMPRSS2* genes (log2 RPM values) corresponding to the cell lines are also shown. At MOI 0.2, the differential patterns in response to SARS-CoV-2 seem somewhat weaker, though largely manifested in these profiles. The SARS-CoV-2 signatures shared across multiple cell lines, as originally identified in A549 with low expression of viral receptor ACE2, is manifested in A549 cells over-expressing ACE2. Interestingly, the Calu-3-specific transcriptional signature, but not our original A549-specific signature, is manifested in A549 over-expressing ACE2. The transcriptional differences between A549 with and without ACE2 would presumably have something to do with ACE2, though it is noted that NHBE also expresses *ACE2* (though at lower levels compared to Calu-3).


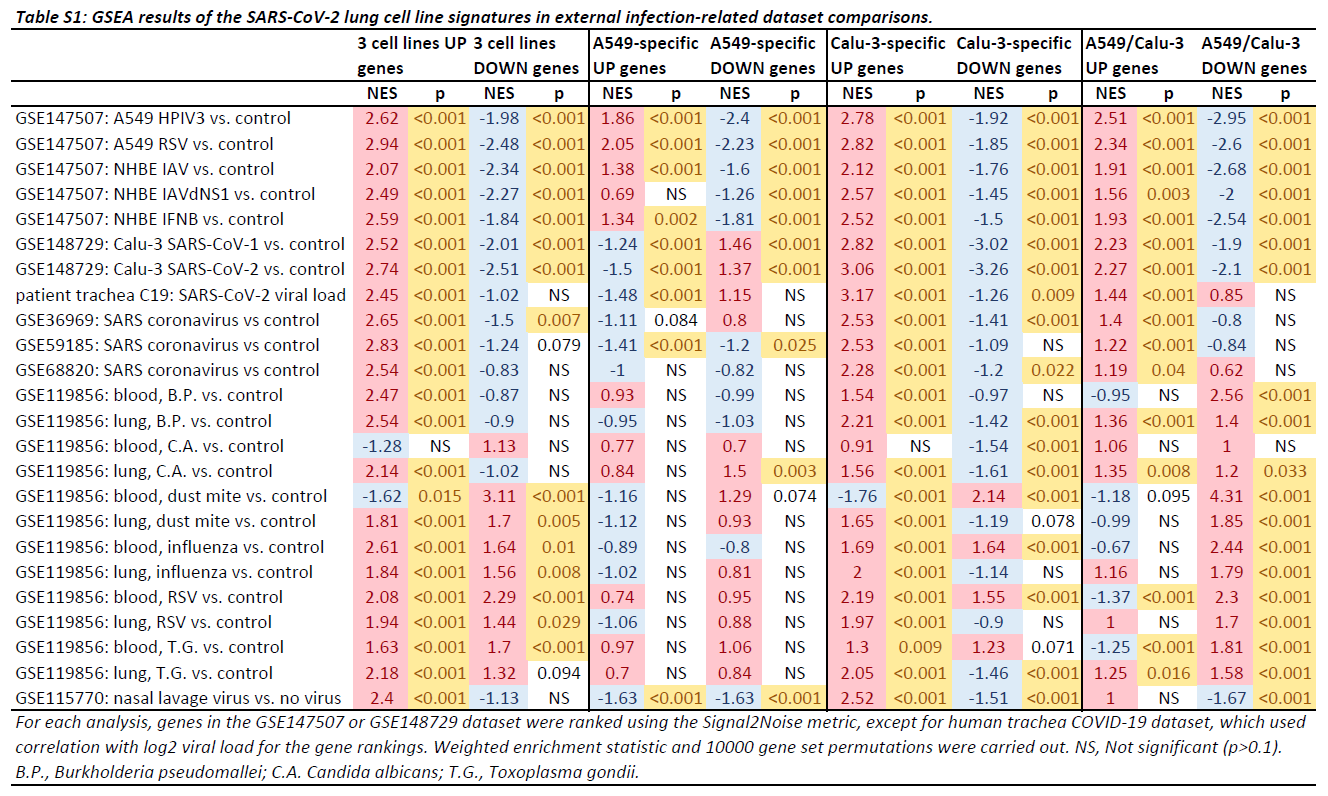


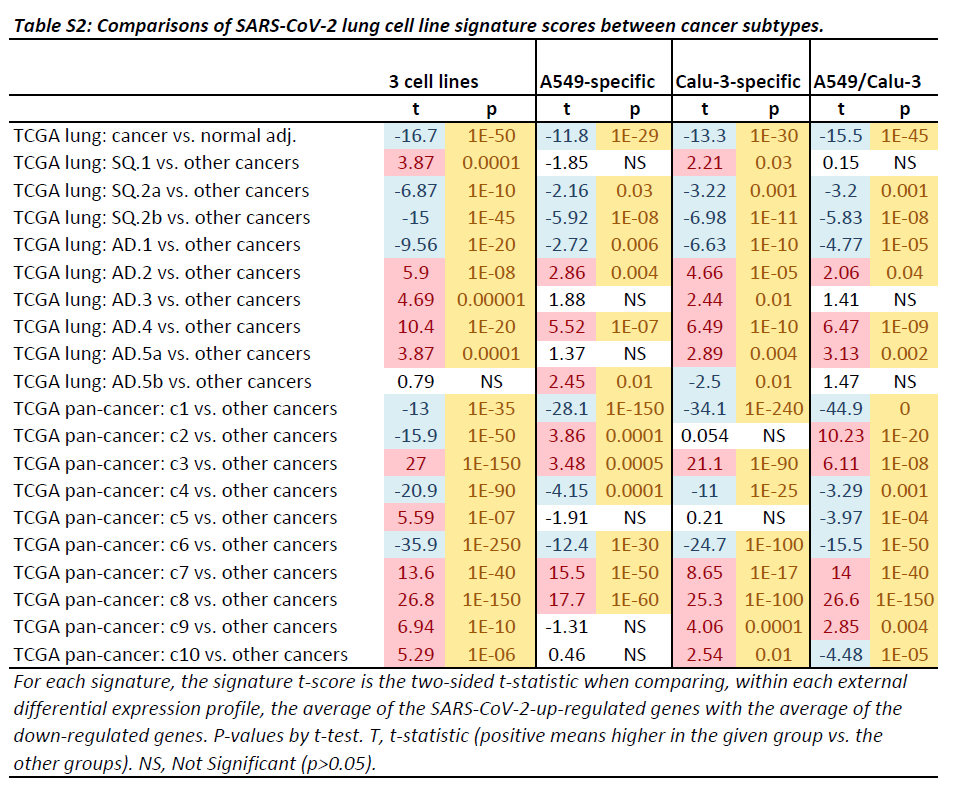

Supplement: Supplementary file 1 — Supplementary Information 1. [file 41598_2021_82221_MOESM1_ESM.docx]
